# Frailty and low utilization of curative-intent surgical resection in nonmetastatic pancreatic cancer

**DOI:** 10.1093/jncics/pkag078

**Published:** 2026-07-31

**Authors:** Lauren Raymond-King, Sean McGrath, Bhramar Mukherjee, Yiran Wang, Harsh Parikh, John Rothen, Pamela R Soulos, Cary P Gross, John W Kunstman

**Affiliations:** Department of Surgery, Division of Surgical Oncology, Yale School of Medicine, New Haven, CT, United States; National Clinician Scholars Program (NCSP), Yale University, New Haven, CT, United States; Department of Biostatistics, Yale School of Public Health, New Haven, CT, United States; Department of Biostatistics, Yale School of Public Health, New Haven, CT, United States; Department of Biostatistics, Yale School of Public Health, New Haven, CT, United States; Department of Biostatistics, Yale School of Public Health, New Haven, CT, United States; Cancer Outcomes, Public Policy, and Effectiveness Research (COPPER) Center, Yale Cancer Center and Yale University School of Medicine, New Haven, CT, United States; Cancer Outcomes, Public Policy, and Effectiveness Research (COPPER) Center, Yale Cancer Center and Yale University School of Medicine, New Haven, CT, United States; National Clinician Scholars Program (NCSP), Yale University, New Haven, CT, United States; Cancer Outcomes, Public Policy, and Effectiveness Research (COPPER) Center, Yale Cancer Center and Yale University School of Medicine, New Haven, CT, United States; Department of Internal Medicine, Yale School of Medicine, New Haven, CT, United States; Department of Surgery, Division of Surgical Oncology, Yale School of Medicine, New Haven, CT, United States

## Abstract

**Background:**

Curative-intent treatment for pancreatic ductal adenocarcinoma necessitates major surgery and chemotherapy, yet many patients do not undergo resection. Frailty may contribute to this phenomenon, but its prevalence and association with pancreatectomy in pancreatic ductal adenocarcinoma remains poorly defined. This study describes the distribution of frailty and its association with curative-intent surgery in a nationally representative cohort of patients with nonmetastatic pancreatic ductal adenocarcinoma.

**Methods:**

Using Surveillance, Epidemiology, and End Results–Medicare data (2013-2019), we identified patients aged 66 years and older with nonmetastatic pancreatic ductal adenocarcinoma. Frailty was categorized as nonfrail, prefrail, mildly frail, or moderately to severely frail using the claims-based frailty index over the 12 months preceding diagnosis. We evaluated frailty distributions via kernel density estimation. We assessed the association between frailty category and receipt of surgery via logistic regression.

**Results:**

Among 8237 patients (mean age = 78 years, 57.6% female, 77.4% White race), 2486 (30.2%) underwent curative-intent pancreatectomy. Surgery was performed in 39.7% of nonfrail and 30.7% of prefrail patients; after adjustment, prefrail patients had similar odds of resection compared to nonfrail patients (adjusted odds ratio [OR] = 1.01, 95% confidence interval [CI] = 0.89 to 1.16). In contrast, 15.8% of mildly frail and 5.7% of moderately to severely frail patients underwent surgery; both groups had statistically significant lower odds of surgery vs nonfrail patients (mildly frail adjusted OR = 0.53, 95% CI = 0.43 to 0.66; moderately to severely frail adjusted OR = 0.18, 95% CI = 0.11 to 0.30).

**Conclusions:**

Remarkably, less than 40% of nonfrail patients with nonmetastatic pancreatic ductal adenocarcinoma underwent surgery, suggesting that curative-intent treatment remains infrequent even among lower-risk adults. Frailty is also common and strongly associated with decreased odds of surgical resection.

## Introduction

Pancreatic ductal adenocarcinoma was the third leading cause of cancer-related deaths in the United States in 2024.^1^ Pancreatic ductal adenocarcinoma is common in patients of advanced age, as the median age at diagnosis is 70 years.^2^ Older adults are a heterogeneous population with varying comorbid conditions, and chronologic age alone is insufficient in explaining differences in treatment selection and outcomes among patients with pancreatic ductal adenocarcinoma. Frailty is a measure that attempts to describe the heterogeneity of aging by evaluating a patient’s physiologic reserve and ability to resist environmental stressors.^3^ It is a superior predictor of morbidity and mortality compared with chronological age alone.^4^ With respect to cancer-related outcomes, frailty is associated with increased mortality, chemotherapy intolerance, and treatment-related toxicity across a wide range of malignancies.^5-7^ In patients with pancreatic ductal adenocarcinoma, the relation between frailty and receipt of curative-intent treatment remains unexplored at the population level. This knowledge gap is noteworthy given the considerable toxicity and risks associated with many pancreatic ductal adenocarcinoma treatment strategies, including major abdominal surgery.

For patients with nonmetastatic pancreatic ductal adenocarcinoma, the current standard of care and optimal treatment strategy is surgery in combination with multiagent systemic chemotherapy.^8-10^ Advances in surgical techniques and the evolution of chemotherapy administration have broadened the patient population that is offered a potentially curative approach to treatment. However, although some older adults can safely undergo pancreatectomy, increased age is associated with a higher risk of postoperative morbidity and mortality.^11^^,^^12^ Regardless of age, population-based studies have demonstrated that in patients with resectable pancreatic ductal adenocarcinoma, more than one-third of patients will never undergo surgery.^13^^,^^14^ Historically, landmark clinical trials for treatment of pancreatic ductal adenocarcinoma have also excluded frail patients.^15-17^ Therefore, it remains unclear how frequently patients with higher frailty undergo curative-intent surgical resection in the real world.

The gold standard for frailty assessment involves a comprehensive clinical evaluation, yet the development of validated and broadly accessible metrics to assess patient frailty in claims data has enabled population-level analyses of frailty and frailty-related outcomes. The claims-based frailty index is a widely used measure that implements a deficit accumulation approach to quantify patient vulnerability by generating a score from 0 to 1 (higher score reflects greater frailty) and represents the culmination of numerous health deficits across multiple domains. The claims-based frailty index has outperformed other indices in measuring the extent of patient frailty not explained by age and sex alone.^18^ The claims-based frailty index has been extensively validated and frequently used in cancer-related research.^19-22^ Using the National Cancer Institute’s Surveillance, Epidemiology, and End Results (SEER)–Medicare linked claims database, the objective of this study is to describe the distribution of frailty in the nonmetastatic pancreatic ductal adenocarcinoma population and to evaluate the association between frailty as defined by the claims-based frailty index and receipt of curative-intent pancreatic resection. These data will provide a better understanding of the prevalence and associations between frailty and receipt of curative-intent therapy, providing population-level insight into how physiologic vulnerability shapes treatment patterns in older adults with nonmetastatic pancreatic ductal adenocarcinoma.

## Methods

### Study design and setting

We performed a quantitative retrospective cohort analysis using SEER-Medicare linked data. The Yale human investigation committee determined that this study did not constitute human participants research.

### Study sample

Patients were included if they were Medicare beneficiaries who were diagnosed with pancreatic ductal adenocarcinoma from 2013 to 2019, aged 66 years or older at diagnosis, and enrolled in continuous Medicare Parts A, B, and D fee-for-service coverage from 1 year prior to diagnosis through end-of-life or 1 year after diagnosis, whichever came first. Patients with metastatic disease at diagnosis; missing stage or sociodemographic information; another cancer diagnosis in the 2 years preceding their pancreatic ductal adenocarcinoma diagnosis; a prior history of metastatic cancer; treatment with radiotherapy or chemotherapy in the year prior to pancreatic ductal adenocarcinoma diagnosis; unknown month of pancreatic ductal adenocarcinoma diagnosis; or diagnosis by autopsy or death certificate were excluded (Figure S1).

### Study variables

The primary study outcome was receipt of curative-intent surgery in the 12-month period following pancreatic ductal adenocarcinoma diagnosis. Curative-intent surgery was defined as partial, distal, or near total pancreatectomy (Current Procedural Terminology [CPT] codes: 48140-48146), pancreaticoduodenectomy (CPT codes: 48150, 48152, 48153, 48154), or total pancreatectomy (CPT codes: 48155-48160). Frailty was defined using the claims-based frailty index, which utilizes Medicare claims data in the year prior to diagnosis to generate a continuous score using a deficit accumulation frailty index model.^23^ The claims-based frailty index was developed and validated as a predictor of death, disability, recurrent falls, and health-care utilization using the Medicare Current Beneficiary Survey in 2006. The claims-based frailty index is calculated by assigning predefined weights to *International Classification of Diseases* diagnosis codes, CPT codes, and Healthcare Common Procedure Coding System procedure codes.

Based on prior studies, we stratified patients into 4 groups based on their prediagnosis claims-based frailty index: nonfrail (claims-based frailty index < 0.15), prefrail (claims-based frailty index ≥ 0.15 and < 0.25), mildly frail (claims-based frailty index ≥ 0.25 and < 0.35), and moderately to severely frail (claims-based frailty index ≥ 0.35).^24^ Lower claims-based frailty index categories reflect less claims-based evidence of physiologic vulnerability and functional impairment, while higher categories reflect increasing accumulation of health deficits.^23^^,^^25^

Patient characteristics included age, sex, race and ethnicity, area-level median household income quintile, Elixhauser Comorbidity Index, tumor location, T staging, and year of diagnosis. Clinical characteristics for descriptive purposes only included number of lymph nodes evaluated, number of positive lymph nodes, N staging, receipt of chemotherapy, and receipt of radiotherapy. For T staging, the groups were divided into T0-T3 tumors vs T4 tumors as a T4 tumor was considered a surrogate for locally advanced pancreatic ductal adenocarcinoma with extrapancreatic extension and/or vascular involvement.^26^

### Statistical analyses

We used kernel density estimation to assess the distribution of continuous claims-based frailty index scores across the total study population and subpopulations defined by surgical status. We evaluated differences in patient characteristics across the 4 claims-based frailty index categories (nonfrail, prefrail, mildly frail, moderately to severely frail) using χ^2^ tests for categorical variables and one-way analysis of variance for continuous variables. We assessed treatment pattern differences using descriptive analyses only (χ^2^ tests) including receipt of chemotherapy (yes or no) and receipt of radiotherapy (yes or no) across the 4 claims-based frailty index categories.

We fit a logistic regression model to examine the unadjusted association between claims-based frailty index category (as independent variable) and surgical resection (as outcome) in patients with pancreatic ductal adenocarcinoma. We then fit a multivariable logistic regression model with robust standard errors to evaluate the relation between the primary independent variable of interest (claims-based frailty index category) and the receipt of surgery, adjusting for clinical and sociodemographic characteristics. Logistic regression model results are presented as odds ratios (ORs) with corresponding 95% confidence intervals (CIs) for the association between claims-based frailty index category and receipt of surgery. Additionally, individual predicted probabilities of surgery were estimated from the multivariable model using observed covariate values and plotted across claims-based frailty index categories.

The regression model was examined for collinearity by assessing variance inflation factors. Covariates with variance inflation factors greater than 3 were considered moderately collinear and evaluated further for model inclusion.^27^ We also performed several sensitivity analyses to account for assumptions made during the regression analyses. We performed a series of regression analyses based on all possible combinations of the 8 adjustment covariates in the model (ie, 256 combinations). We also calculated the E-values for the adjusted odds ratio estimates and confidence intervals between each of the claims-based frailty index categories and surgical resection, which quantify how strong an unmeasured variable would need to be associated with the claims-based frailty index category and surgery to nullify the claims-based frailty index category–surgery association.^28^ Last, we fit unadjusted and adjusted logistic regression models for surgery where claims-based frailty index was treated as a continuous variable and report odds ratios corresponding to 1 interquartile range (IQR) change in claims-based frailty index. All analyses were conducted using STATA version 18.0 and R version 4.3.1.

## Results

Our study sample included 8237 patients (Figure S1). The median age was 78 years, and most patients were female (57.6%) and White race (77.4%) and had tumors in the head of the pancreas (63.3%) (Table 1). There were 2585 (31.4%) nonfrail patients, 4043 (49.1%) prefrail patients, 1256 (15.2%) mildly frail patients, and 353 (4.3%) moderately to severely frail patients in the total cohort with a median claims-based frailty index score of 0.18.

**Table 1.** Sociodemographic and clinical characteristics of patients with nonmetastatic pancreatic ductal adenocarcinoma (*n* = 8237), by CFI category.^a^

Characteristic	Full sample, No. (%)^b^	CFI frailty strata				
		Nonfrail CFI 1, No. (%)	Prefrail CFI 2, No. (%)	Mildly frail CFI 3, No. (%)	Moderately to severely frail CFI 4, No. (%)	*P*
Total	8237	2585	4043	1256	353	
Age, median (IQR), y	78 (72-84)	75 (70-80)	78 (73-84)	81 (75-87)	83 (76-88)	<.001*
Gender						<.001*
Male	3489 (42.4)	1272 (36.4)	1695 (48.6)	418 (12.0)	104 (3.0)	
Female	4748 (57.6)	1313 (27.7)	2348 (49.5)	838 (17.6)	249 (5.2)	
Race and ethnicity						<.001*
Asian	479 (5.8)	169 (35.3)	240 (50.1)	56 (11.7)	14 (2.9)	
Black	581 (7.1)	123 (21.2)	289 (49.7)	118 (20.3)	51 (8.8)	
Hispanic	618 (7.5)	180 (29.1)	297 (48.1)	108 (17.5)	33 (5.3)	
White	6376 (77.4)	<2042 (<32.0)	3139 (49.2)	951 (14.9)	>244 (>3.8)	
Unknown	183 (2.2)	>71 (>38.8)	78 (42.6)	23 (12.6)	<11 (<6.0)	
Area-level median household income						<.001*
Lowest quintile	1630 (19.8)	397 (24.3)	829 (50.9)	305 (18.7)	99 (6.1)	
Second quintile	1663 (20.2)	458 (27.5)	860 (51.7)	271 (16.3)	74 (4.5)	
Middle quintile	1587 (19.3)	520 (32.8)	749 (47.2)	243 (15.3)	75 (4.7)	
Fourth quintile	1668 (20.2)	566 (33.9)	810 (48.6)	242 (14.5)	50 (3.0)	
Highest quintile	1689 (20.5)	644 (38.1)	795 (47.1)	195 (11.5)	55 (3.3)	
Elixhauser Comorbidity Index						<.001*
0 conditions	2150 (26.1)	>1482 (>68.9)	617 (28.7)	40 (1.9)	<11 (<0.5)	
1-2 conditions	3324 (40.4)	<972 (<29.2)	2046 (61.5)	288 (8.7)	>18 (>0.5)	
≥3 conditions	2763 (33.5)	131 (4.7)	1380 (50.0)	928 (33.6)	324 (11.7)	
Tumor location						
No surgery						<.001*
Head	3497 (60.8)	984 (28.2)	1704 (48.7)	620 (17.7)	189 (5.4)	
Body, tail	878 (15.3)	276 (31.4)	439 (50.0)	126 (14.4)	37 (4.2)	
Other	1376 (23.9)	<302 (21.9)	660 (48.0)	311 (22.6)	>103 (>7.5)	
Surgery						.033*
Head	1720 (69.2)	733 (42.6)	853 (49.6)	121 (7.0)	13 (0.8)	
Body, tail	510 (20.5)	>192 (>37.6)	253 (49.6)	54 (10.6)	<11 (<2.2)	
Other	256 (10.3)	98 (38.3)	134 (52.3)	24 (9.4)	0 (0)	

Abbreviation: CFI = claims-based frailty index.

a χ^2^ tests were used for categorical variables, and 1-way analysis of variance was used for age (continuous variable).

b Percentages in the CFI frailty strata represent row percentages. Percentages in the full sample column represent the proportion of the total cohort.

* *P* < .05 is statistically significant.

The distribution of claims-based frailty index scores differed between the surgical and nonsurgical populations (Figure 1). Surgical patients tended toward lower claims-based frailty index scores with a median of 0.16. In contrast, nonsurgical patients had a median claims-based frailty index of 0.19 and a heavier right tail toward higher frailty. The surgical patients also had a statistically significant lower mean claims-based frailty index score than the nonsurgical patients (0.17 vs 0.20, respectively; *P* < .001).

**Figure 1.**
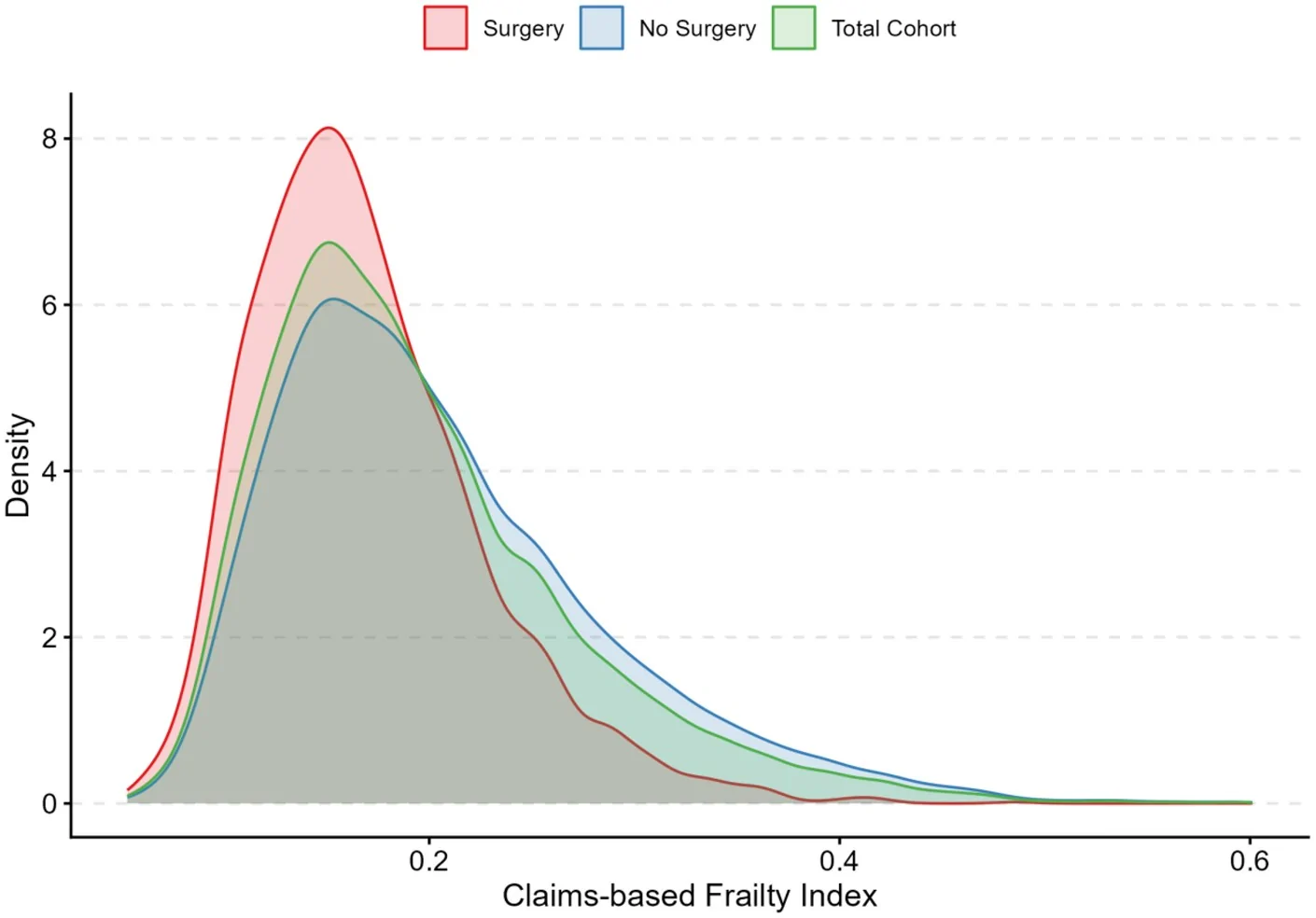
Distribution of frailty using the claims-based frailty index, by surgery status, in nonmetastatic pancreatic ductal adenocarcinoma. This figure shows the kernel density estimate of the distribution of the claims-based frailty index among individuals who underwent surgery (n = 2486), individuals who did not undergo surgery (n = 5751), and the total cohort (n = 8237).

There were statistically significant differences in patients’ sociodemographic characteristics across claims-based frailty index categories, including age, sex, race and ethnicity, median income quintile, Elixhauser Comorbidity Index, and tumor location (Table 1). Older patients, females, patients of Black race, patients residing in a lower median income region, and those with more medical comorbidities were more frequently moderately to severely frail.

### Treatment patterns stratified by frailty

Curative-intent surgical resection was performed in 2486 (30.2%) patients. The nonfrail and prefrail categories had a higher proportion of patients undergoing curative surgery than those in the mildly frail or moderately to severely frail categories (Figure 2A). A similar pattern was observed for receipt of chemotherapy or radiotherapy, with increasing frailty resulting in a lower percentage of patients receiving either treatment. Regarding combined treatments, most patients (79.5%) who underwent curative-intent surgery received at least 1 cycle of chemotherapy, regardless of frailty (Figure 2B). However, receipt of chemotherapy in addition to surgery differed significantly across frailty categories, with lower percentages observed among increasingly frail groups (*P* < .001).

**Figure 2.**
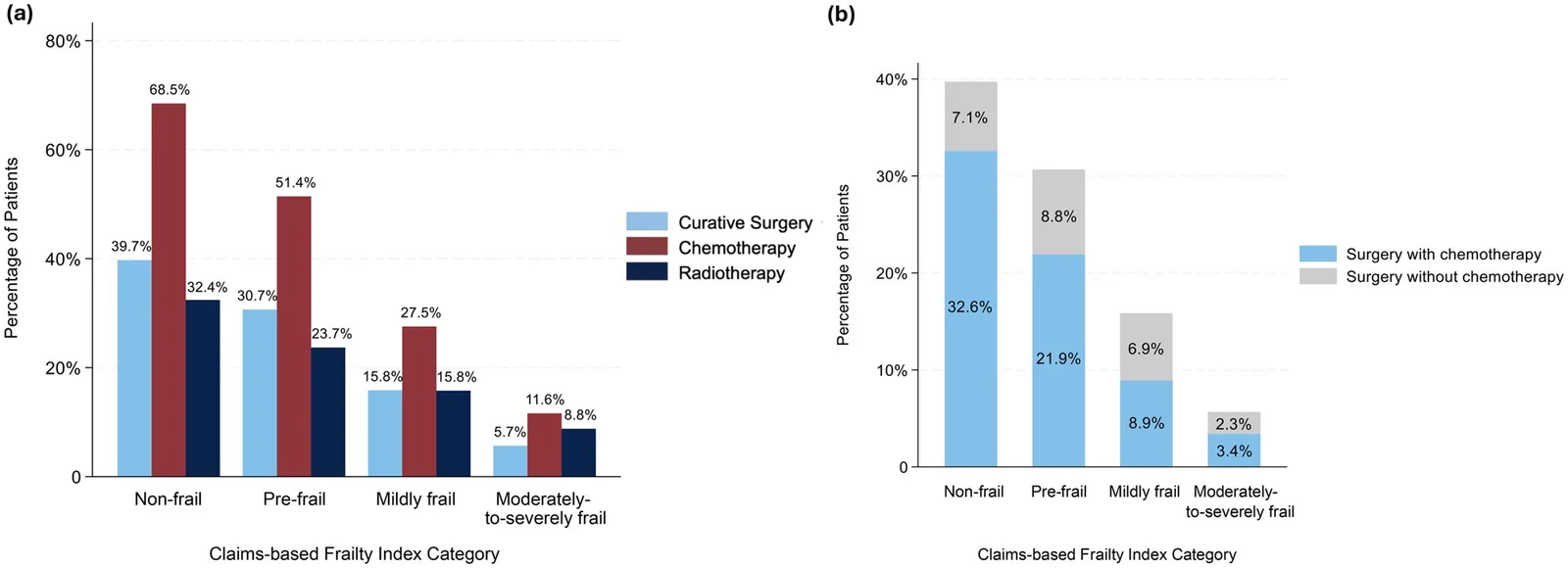
Treatment characteristics of nonmetastatic pancreatic ductal adenocarcinoma patients, by claims-based frailty index category. **A**) Demonstrates the percentage of patients within each frailty stratum who underwent curative surgery, chemotherapy, or radiotherapy. **B**) Stacked bar graph that represents all patients within each frailty stratum who underwent curative surgery, stratified by receipt of chemotherapy.

Tumor characteristics did not vary across the 4 frailty categories in those who underwent curative-intent pancreatic resection. Most patients had a T3 tumor, were N0 or N1, and had more than 10 lymph nodes examined at the time of surgery (Table S1). Across all frailty categories, 18.2% of patients had T4 tumors, also described as locally advanced tumors, indicating involvement of major adjacent vascular structures. The prevalence of T4 tumors did not vary across frailty groups, but the proportion undergoing surgery differed. Of the T4 patients, 11.6% underwent curative-intent surgery, and 95.6% of these surgical patients were either nonfrail or prefrail.

### Association between frailty and receipt of surgery

Higher claims-based frailty index category was associated with a statistically significant decrease in the odds of receiving curative-intent pancreatic resection compared to nonfrail patients (prefrail: OR = 0.67, 95% CI = 0.61 to 0.74; mildly frail: OR = 0.29, 95% CI = 0.24 to 0.34; moderately to severely frail: OR = 0.09, 95% CI = 0.06 to 0.14) (Table 2).

**Table 2.** Logistic regression models evaluating the relation between frailty and receipt of surgery.

	Bivariate		Multivariable	
Variable	OR (95% CI)	*P*	aOR (95% CI)	*P*
Frailty category				
Nonfrail	1.00 (Referent)	—	1.00 (Referent)	—
Prefrail	0.67 (0.61 to 0.74)	<.001*	1.01 (0.89 to 1.16)	.833
Mildly frail	0.29 (0.24 to 0.34)	<.001*	0.53 (0.43 to 0.66)	.001*
Moderately to severely frail	0.09 (0.06 to 0.14)	<.001*	0.18 (0.11 to 0.30)	<.001*
Continuous CFI^a^	0.50 (0.46 to 0.53)	<.001*	0.62 (0.56 to 0.68)	<.001*

Abbreviations: aOR = adjusted odds ratio; CFI = claims-based frailty index robust standard errors used in the multivariable model; CI = confidence interval; OR = odds ratio.

a Odds ratios for continuous variables are expressed per 1 interquartile range increase in the CFI score.

* *P* < .05 is statistically significant.

After adjusting for clinical and sociodemographic characteristics, patients in the prefrail category had similar odds of undergoing surgical resection compared with nonfrail patients (adjusted OR = 1.01, 95% CI = 0.89 to 1.16) (Table 2; see also Figure S2). Patients in the mildly frail and moderately to severely frail categories, however, had statistically significant decreased odds compared with those in the nonfrail category of undergoing surgical resection (mildly frail: adjusted OR = 0.53, 95% CI = 0.43 to 0.66; moderately to severely frail: adjusted OR = 0.18, 95% CI = 0.11 to 0.30). The median of the estimated probabilities of surgery was 0.44 (IQR = 0.20-0.57) among nonfrail patients, 0.29 (IQR = 0.11-0.48) in prefrail, 0.12 (IQR = 0.04-0.26) in mildly frail, and 0.03 (IQR = 0.01-0.10) in moderately to severely frail (Figure 3). Covariates in the adjusted model that were associated with a decreased odds of surgery included increased age, Black race, having at least 3 comorbid conditions, and T4 stage (Table S2). There was no statistically significant interaction between age and frailty when evaluating their association with receipt of surgery. Alternatively, covariates associated with an increased odds of surgery included a higher area-level median household income and a body and/or tail pancreatic tumor.

**Figure 3.**
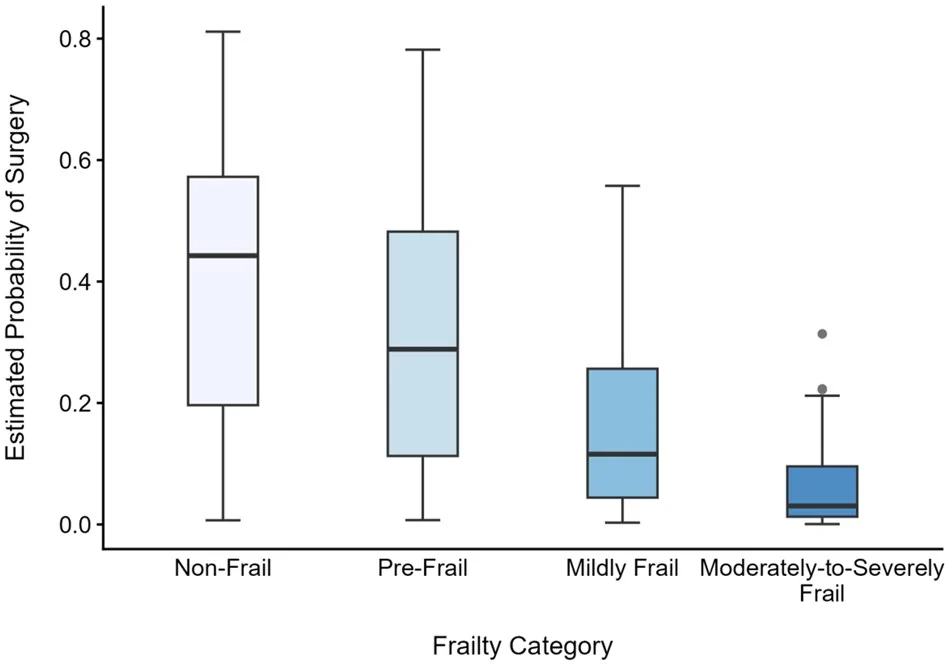
Distribution of estimated probabilities of surgery by frailty category. The box plots show the estimated probabilities of surgery among individuals in each frailty group: nonfrail (n = 2585), prefrail (n = 4043), mildly frail (n = 1256), and moderately to severely frail (n = 353). The **boxes** represent the first and third quartiles with a horizontal line at the median. The **whiskers** extend to the largest value within 1.5 times the interquartile range from the box. Values beyond the whiskers are shown as individual points. The estimated probabilities were obtained from the multivariable logistic regression model.

All variance inflation factors in the adjusted multivariable logistic regression model were less than 3, indicating no substantial multicollinearity. When removing the Elixhauser Comorbidity Index as a covariate, the model again demonstrated a significantly lower odds of surgery for mildly frail (adjusted OR = 0.44, 95% CI = 0.36 to 0.53) and moderately to severely frail (adjusted OR = 0.15, 95% CI = 0.09 to 0.23) patients compared with nonfrail patients with no statistically significant association with receipt of surgery for prefrail patients (adjusted OR = 0.91, 95% CI = 0.81 to 1.02).

The results of the sensitivity analyses including each possible choice of adjustment covariates in the model are shown in Figure S3. The adjusted odds ratios and corresponding 95% confidence intervals for the mildly frail and moderately to severely frail categories were always below 1.0, whereas the adjusted odds ratios and 95% confidence intervals for the prefrail category included 1.0 for certain combinations of covariates. The E-values for the adjusted odds ratio estimates in the adjusted model were 1.09 for the prefrail, 2.08 for the mildly frail, and 4.09 for the moderately to severely frail.

When treating claims-based frailty index as a continuous variable in the logistic regression analyses, the adjusted odds ratio for surgery was 0.62 (95% CI = 0.56 to 0.68) for 1 IQR increase in the claims-based frailty index, that is, a change from 0.14 (25th percentile) to 0.24 (75th percentile) in the claims-based frailty index.

## Discussion

In our study of 8237 patients with nonmetastatic pancreatic ductal adenocarcinoma, 68.6% of patients were found to have some degree of frailty, while only 31.4% of patients were classified as nonfrail. The proportion of frail patients in this nonmetastatic pancreatic ductal adenocarcinoma population is much higher than that of the general Medicare population (52.7% nonfrail, 47.3% prefrail or higher).^25^ At the population level, frailty had a substantial negative association with receipt of surgical resection. However, even when limited to nonfrail patients, only 40% underwent surgery. Moreover, in those who did undergo surgical resection, fewer patients received systemic chemotherapy as frailty increased.

Frailty is an aging-related syndrome that can exert a profound effect on each stage of the care continuum for patients with cancer. While frailty metrics are beginning to emerge in clinical research, many clinical trials still utilize performance status alone as a surrogate for health status. The landmark clinical trials for pancreatic ductal adenocarcinoma that established the current standard of care for optimal treatment have required patients to have an Eastern Cooperative Oncology Group Performance Status of 0-1 (fully active to restricted in strenuous activity only) or Karnofsky Performance Status of at least 50% (functionally capable and able to care for most personal needs) to be eligible for enrollment.^15-17^^,^^29-31^

It is recognized that clinical trial data do not adequately represent the general population, nor are the results generalizable to the real world.^32^ In this study of a representative and real-world population of Medicare recipients in the United States, only 31.4% of patients were classified as nonfrail and therefore potentially able to qualify for clinical trial enrollment. The largest subpopulation identified in this study was prefrail patients. These patients have claims suggesting emerging deficits, such as a history of falls, mild cognitive decline, or early dependency. When combined with the frail population, these data suggest that as many as two-thirds of nonmetastatic pancreatic ductal adenocarcinoma patients are not represented by the typical clinical trial population. Thus, it is difficult to decipher the optimal modality of treatment for these patients as well as the expected outcomes of these treatments.

Advances in surgical technique and preoperative patient optimization have enabled curative-intent surgical resection to be offered more widely. It is striking, therefore, that only 30.2% of patients underwent curative-intent surgery in this nonmetastatic population. Even when limited to nonfrail patients with tumors lacking extrapancreatic extension or arterial invasion (T0-T3), only 45.2% underwent surgical resection. Although this subpopulation does not rule out venous involvement, this group most closely approximates patients considered “resectable” or “borderline resectable” by the current National Comprehensive Cancer Network guidelines with the most favorable survival following surgery and chemotherapy. Notably, nearly 20% of surgical patients in this group did not receive a single cycle of chemotherapy. These data suggest that adherence to treatment guidelines for optimal outcomes in patients with nonmetastatic pancreatic ductal adenocarcinoma is imperfect at best.

We found that, after adjusting for sociodemographic and clinical characteristics, prefrail patients had similar odds of surgical resection compared with nonfrail patients, while mildly or moderately to severely frail patients continued to have substantially decreased odds of surgical resection. Several studies have evaluated the effect of chronological age on postoperative outcomes and found that advanced age is associated with a statistically significant higher risk of postoperative morbidity and mortality compared with younger patients but that surgery could safely be accomplished in select patients.^11^^,^^12^^,^^33^ This population is often the target for prehabilitation programs prior to surgery to improve physical function and functional capacity.^34^^,^^35^ In aggregate, these data suggest that surgeons were amenable to offering surgery to prefrail patients if, after comprehensive evaluation, the risk of operating could be favorably modified or addressed.

A major novel finding of this study is that regardless of frailty, only a small proportion of patients received curative-intent treatment for nonmetastatic pancreatic ductal adenocarcinoma. Existing studies have highlighted the low incidence of surgery in patients with pancreatic ductal adenocarcinoma, attributing this finding to advanced disease stage and patient characteristics such as age or frailty.^14^^,^^36^^,^^37^ In this study, we conclusively demonstrate that even in candidates seemingly best suited for curative-intent resection (nonmetastatic and nonfrail), surgery is still surprisingly infrequent. Further research is needed to understand the reason for this finding, which is likely multifactorial.

In addition to patient- and tumor-level characteristics, treatment selection is likely influenced by unmeasured provider- and system-level determinants. For instance, in this study, we found that patients residing in a region with a higher median income had higher odds of undergoing surgical resection, demonstrating the interplay of underlying socioeconomic factors with patient care. Prior work has also demonstrated substantial geographic- and hospital-level variation in patient access to curative-intent resection, suggesting that referral patterns, institutional practice variations, and multidisciplinary treatment pathways may contribute to the observed differences in treatment utilization.^38^ At the individual level, decisions regarding curative-intent surgery are complex and may be influenced by surgeon experience, institutional support for high-risk operative management, rehabilitation resources, caregiver support, and patient preferences. Although unmeasured confounding remains possible, sensitivity analyses using E-values suggested that progressively stronger unmeasured confounding would be required to fully explain the observed associations between higher frailty categories and lower odds of surgical resection (Figure S3).^28^ Moreover, these findings were not limited to surgery because receipt of systemic therapy and radiation were also much lower than expected and decreased across increasing frailty categories.

There are several limitations of this study. This analysis was performed on observational, retrospective data and is therefore limited by selection bias and retrospectively defined parameters. For example, SEER-Medicare does not capture data such as radiographic findings, medical decision-making, and intent of treatment. Additionally, although SEER-Medicare covers a large portion of the US population, it does not represent all regions or demographic groups. However, claims-based datasets such as SEER-Medicare more accurately capture treatment data compared with other widely used oncology datasets such as the National Cancer Database and are superior at capturing comorbid conditions that constitute the basis of the frailty index.^39^^,^^40^

In summary, frailty is a statistically significant factor associated with lower odds of curative-intent surgical resection in patients with nonmetastatic pancreatic ductal adenocarcinoma. With more than two-thirds of the nonmetastatic pancreatic ductal adenocarcinoma population falling into at least a prefrail category, it is imperative to understand how frailty is associated with treatment patterns and outcomes. There remains a substantial void in the literature around frailty and its association with cancer-directed treatment and clinical outcomes in patients with localized pancreatic ductal adenocarcinoma. Future work will focus on addressing this knowledge gap by analyzing patterns of care, clinical outcomes of specific treatment choices, and overall survival in this patient population.

## Supplementary Material

pkag078_Supplementary_Data

## Data Availability

The data underlying this article were provided by SEER-Medicare by permission via a data use agreement. Data will be shared on request to the corresponding author with permission of SEER-Medicare.
