# Supplementary material for: Frailty and low utilization of curative-intent surgical resection in nonmetastatic pancreatic cancer
Source: JNCI Cancer Spectr. 2026 Jul 31;10(5):pkag078. doi: 10.1093/jncics/pkag078 (PMC13637609; doi:10.1093/jncics/pkag078)
Supplement: Supplementary file 1 [file pkag078_supplementary_data.zip › Appendix 5.docx]

**Figure S3: Sensitivity analyses using multiple regression analyses based on all possible combinations of the eight adjustment covariates for each frailty category**

Figure S3A: The adjusted odds ratio between pre-frail patients (reference: non-frail patients) and surgical resection, adjusting for all possible combinations of the following eight adjustment covariates: age (66-69, 70-74, 75-79, 80-84, 85+), sex (male, female), race/ethnicity (White, Black, Hispanic, Asian, unknown), median income quintile, Elixhauser Comorbidity Index (0, 1-2, 3+), tumor location (head, body/tail, other), T staging (T0-T3, T4), and year of diagnosis. The E-value for the estimated odds ratio between pre-frail patients and surgical resection in the fully adjusted model is computed.

Figure S3B: The adjusted odds ratio between mildly frail patients (reference: non-frail patients) and surgical resection, adjusting for all possible combinations of the following eight adjustment covariates: age (66-69, 70-74, 75-79, 80-84, 85+), sex (male, female), race/ethnicity (White, Black, Hispanic, Asian, unknown), median income quintile, Elixhauser Comorbidity Index (0, 1-2, 3+), tumor location (head, body/tail, other), T staging (T0-T3, T4), and year of diagnosis. The E-value for the estimated odds ratio between mildly frail patients and surgical resection in the fully adjusted model is computed.

Figure S3C: The adjusted odds ratio between moderately-to-severely frail patients (reference: non-frail patients) and surgical resection, adjusting for all possible combinations of the following eight adjustment covariates: age (66-69, 70-74, 75-79, 80-84, 85+), sex (male, female), race/ethnicity (White, Black, Hispanic, Asian, unknown), median income quintile, Elixhauser Comorbidity Index (0, 1-2, 3+), tumor location (head, body/tail, other), T staging (T0-T3, T4), and year of diagnosis. The E-value for the estimated odds ratio between moderately-to-severely frail patients and surgical resection in the fully adjusted model is computed.
