# Supplementary material for: Frailty and low utilization of curative-intent surgical resection in nonmetastatic pancreatic cancer
Source: JNCI Cancer Spectr. 2026 Jul 31;10(5):pkag078. doi: 10.1093/jncics/pkag078 (PMC13637609; doi:10.1093/jncics/pkag078)
Supplement: Supplementary file 1 [file pkag078_supplementary_data.zip › Appendix 1.docx]

**Figure S1: Flow diagram of patient selection for non-metastatic pancreatic cancer**

Pancreatic cancer diagnosis from 2013-2019 **(n = 106,363)**

Patients excluded **(n = 88,098)**

Histology inconsistent with pancreatic ductal adenocarcinoma (PDAC) (n = 7,755)

Identified at autopsy or on death certificate (n = 5,066)

Without continuous Medicare Part A, B, or D coverage from 1 year prior to diagnosis to 1 year after diagnosis (or death if earlier) (n = 31,895)

No age at diagnosis (n = 620)

<66 years old at diagnosis (n = 12,669)

Expansion registries including NY, MA, ID, and TX due to missing tumor and stage information (n = 30,093)

**Identification**

Remaining patients **(n = 18,265)**

Patients excluded **(n = 10,028)**

Another cancer diagnosis 2 years before PDAC diagnosis, prior diagnosis of metastatic cancer, and radiotherapy or chemotherapy in the year before PDAC diagnosis (n = 1,429)

M1 disease (n = 4,898)

Missing M stage (n = 3,541)

Missing sociodemographic information (n = 160)

**Clinical and Sociodemographic Information**

Final cohort **(n = 8,237)**

**Included**
