# Supplementary material for: Frailty and low utilization of curative-intent surgical resection in nonmetastatic pancreatic cancer
Source: JNCI Cancer Spectr. 2026 Jul 31;10(5):pkag078. doi: 10.1093/jncics/pkag078 (PMC13637609; doi:10.1093/jncics/pkag078)
Supplement: Supplementary file 1 [file pkag078_supplementary_data.zip › Appendix 2.docx]

**Table S1: Tumor characteristics of patients with non-metastatic PDAC who underwent surgical resection, by CFI category. Chi-squared tests were used to determine p-values.**

|  | Received Surgery | | | |
| --- | --- | --- | --- | --- |
| **Tumor Characteristics** | Non-frail  CFI 1  n (%)  N = 1027 | Pre-frail  CFI 2  n (%)  N = 1240 | Mildly frail  CFI 3  n (%)  N = 199 | p-value |
| Number of Lymph Nodes Evaluated |  |  |  | 0.033* |
| 0 | 110 (10.7) | 123 (9.9) | 22 (11.1) |  |
| 1-5 | 48 (4.7) | 87 (7.0) | 17 (8.5) |  |
| 6-10 | 98 (9.5) | 147 (11.9) | 30 (15.1) |  |
| 11-15 | 195 (19.0) | 234 (18.9) | 31 (15.6) |  |
| 16-20 | 193 (18.8) | 226 (18.2) | 42 (21.1) |  |
| >20 | 371 (36.1) | 412 (33.2) | 57 (28.6) |  |
| Not reported | 12 (1.2) | 11 (0.9) | 0 (0) |  |
| Number of Positive Lymph Nodes |  |  |  | 0.939 |
| 0 | 483 (47.0) | 571 (46.0) | 90 (45.2) |  |
| 1-3 | 336 (32.7) | 404 (32.6) | 70 (35.2) |  |
| 4-6 | 106 (10.3) | 153 (12.3) | >17 (>8.5) |  |
| 7-10 | >55 (>5.5) | >68 (>5.5) | <11 (<5.5) |  |
| >10 | 36 (3.5) | 38 (3.1) | <11 (<5.5) |  |
| Not reported | <11 (<1.0) | <11 (<0.5) | 0 (0) |  |
| T stage |  |  |  | 0.098 |
| T0 | 0 (0) | <11 (<0.9) | <11 (<5.5) |  |
| Tis | 29 (2.8) | 50 (4.0) | <11 (<5.5) |  |
| T1 | 49 (4.8) | 82 (6.6) | <11 (<5.5) |  |
| T2 | 32 (3.1) | 62 (5.0) | <11 (<5.5) |  |
| T3 | 593 (57.7) | 675 (54.5) | >84 (>42.2) |  |
| T4 | 77 (7.5) | 74 (6.0) | <11 (<5.5) |  |
| TX | 20 (2.0) | 29 (2.3) | <11 (<5.5) |  |
| Not reported | 227 (22.1) | >257 (>20.7) | 49 (24.6) |  |
| N stage |  |  |  | 0.805 |
| N0 | 448 (43.6) | 532 (42.9) | 91 (45.7) |  |
| N1 | 503 (49.0) | 625 (50.4) | >83 (>41.7) |  |
| N2 | 57 (5.6) | 67 (5.4) | 14 (7.1) |  |
| NX | 19 (1.8) | 16 (1.3) | <11 (<5.5) |  |

PDAC, pancreatic ductal adenocarcinoma; CFI, Claims-based Frailty Index

Moderately-to-severely frail category was not shown due to small cell sizes

*p-value <0.05 is significant
