# Supplementary material for: Frailty and low utilization of curative-intent surgical resection in nonmetastatic pancreatic cancer
Source: JNCI Cancer Spectr. 2026 Jul 31;10(5):pkag078. doi: 10.1093/jncics/pkag078 (PMC13637609; doi:10.1093/jncics/pkag078)
Supplement: Supplementary file 1 [file pkag078_supplementary_data.zip › Appendix 3.docx]

**Figure S2: Logistic regression models evaluating the association between frailty and receipt of surgery**

This figure shows the unadjusted and adjusted odds ratios (ORs) between measures of frailty and surgery. The OR for surgery based on treating the Claims-based Frailty Index (CFI) as a continuous variable corresponds to a one interquartile range increase in the CFI score. The adjustment variables included age (66-69, 70-74, 75-79, 80-84, 85+), sex (male, female), race/ethnicity (White, Black, Hispanic, Asian, unknown), median income quintile, Elixhauser Comorbidity Index (0, 1-2, 3+), tumor location (head, body/tail, other), T staging (T0-T3, T4), and year of diagnosis.
