# Supplementary material for: Frailty and low utilization of curative-intent surgical resection in nonmetastatic pancreatic cancer
Source: JNCI Cancer Spectr. 2026 Jul 31;10(5):pkag078. doi: 10.1093/jncics/pkag078 (PMC13637609; doi:10.1093/jncics/pkag078)
Supplement: Supplementary file 1 [file pkag078_supplementary_data.zip › Appendix 4.docx]

**Table S2: Covariates used in the multivariable logistic regression model evaluating the relation between frailty and receipt of surgery**

| **Variable** | **aOR (95% CI)** | **p-value** |
| --- | --- | --- |
| Age |  |  |
| 66-69 | 1.00 [Ref] | - |
| 70-74 | 0.75 (0.64 – 0.88) | <0.001* |
| 75-79 | 0.61 (0.52 – 0.72) | <0.001* |
| 80-84 | 0.30 (0.25 – 0.36) | <0.001* |
| 85+ | 0.07 (0.06 – 0.09) | <0.001* |
| Gender |  |  |
| Male | 1.00 [Ref] | - |
| Female | 1.00 (0.89 – 1.11) | 0.952 |
| Race |  |  |
| White | 1.00 [Ref] | - |
| Asian | 0.92 (0.73 – 1.16) | 0.503 |
| Black | 0.63 (0.50 – 0.79) | <0.001* |
| Hispanic | 1.00 (0.81 – 1.23) | 0.993 |
| Unknown | 1.32 (0.95 – 1.84) | 0.102 |
| Area-Level Median Household Income |  |  |
| Lowest Quintile | 1.00 [Ref] | - |
| Second Quintile | 1.05 (0.88 – 1.25) | 0.595 |
| Middle Quintile | 1.22 (1.02 – 1.46) | 0.027* |
| Fourth Quintile | 1.51 (1.27 – 1.80) | <0.001* |
| Highest Quintile | 1.99 (1.68 – 2.37) | <0.001* |
| Elixhauser Comorbidity Index |  |  |
| 0 Conditions | 1.00 [Ref] | - |
| 1-2 Conditions | 0.88 (0.77 – 1.01) | 0.069 |
| 3+ Conditions | 0.74 (0.62 – 0.88) | <0.001* |
| Year of Diagnosis | 1.02 (0.99 – 1.04) | 0.219 |
| Tumor Location |  |  |
| Head | 1.00 [Ref] | - |
| Body/tail | 1.24 (1.08 – 1.43) | 0.003* |
| Other | 0.46 (0.39 – 0.54) | <0.001* |
| T Stage |  |  |
| T0-T3 | 1.00 [Ref] | - |
| T4 | 0.17 (0.14 – 0.20) | <0.001* |

*p-value <0.05 is significant
